# Influence of Micro-Nanostructured Anatase-Coated SLA Titanium on Macrophage Behavior

**DOI:** 10.3390/jfb17030111

**Published:** 2026-02-25

**Authors:** Leila Mohammadnejad, Madeline Mangold, Hannah Conrady, Wafa Zafira, Evi Kimmerle-Mueller, Peter Schneider, Barbara Illing, Christiane von Ohle, Annika Hechler, Frank Rupp, Stefanie Krajewski

**Affiliations:** 1 Department of Medical Materials Science & Technology, Institute of Biomedical Engineering, University Hospital Tübingen, Osianderstr. 2-8, 72076 Tübingen, Germany; 2 Department of Conservative Dentistry and Periodontology, University Hospital Tübingen, Osianderstr. 2-8, 72076 Tübingen, Germany

**Keywords:** anatase, immunomodulation, human monocyte-derived macrophages, dental implants

## Abstract

The success of titanium dental implants rely on osseointegration, influenced by surface properties and early immune responses. While sandblasted and acid-etched (SLA) titanium surfaces have shown clinical success, macrophage-mediated immune responses at these interfaces remain poorly understood. Anatase nanostructures have been shown to influence macrophage polarization on smooth titanium, but their effects on micro-rough SLA surfaces are not fully explored. This study investigates the immunomodulatory effects of micro-nanostructured anatase coatings on SLA titanium using human monocyte-derived macrophages (MDMs). M0-MDMs, were cultured and polarized to M1 and M2- macrophages on Ti-machined, Ti-SLA, Ti-SLA-anatase, and coverslip control surfaces for 48 h. Macrophage behavior was assessed using CCK-8 assay, confocal microscopy, SEM, ELISA, and qRT-PCR. All surfaces demonstrated excellent cytocompatibility, with similar macrophage viability across all investigated groups. M1 macrophages showed upregulation of CCR7 and TNF-α, while M2 macrophages expressed CD209 and CCL13 across all surfaces. Importantly, Ti-SLA-anatase did not significantly alter M1 or M2 markers, cytokine secretion, or gene expression, and did not exacerbate inflammatory responses. Micro-nanostructured anatase coatings on SLA titanium are immunologically well-tolerated and do not increase inflammation. These findings, combined with previously reported enhanced osteogenic properties, suggest the clinical potential of anatase-coated SLA surfaces.

## 1. Introduction

Titanium and its alloys are widely employed in dental and orthopedic implants due to their excellent mechanical strength, corrosion resistance, and inherent biocompatibility [1]. The clinical success of these implants fundamentally depends on osseointegration, the direct structural and functional connection between living bone and the implant surface. This critical process is strongly influenced by surface properties, such as surface topography, chemistry, surface energy, and hydrophilicity, making surface modification strategies pivotal for optimizing implant performance [2,3]. Among the various surface treatments available, sandblasted and acid-etched (SLA) titanium has become a widely applied and clinically successful implant surface, as its micro-roughness enhances bone anchorage and long-term stability [4,5]. The micro-topography generated by SLA treatment increases the surface area for protein adsorption and cellular attachment, thereby facilitating superior bone anchorage compared to machined surfaces [6,7]. However, conventional SLA surfaces display some limitations during the early healing phase: their inherently hydrophobic nature has been shown to hinder the initial protein adsorption and cellular attachment, while their micro-rough topography is prone to bacterial colonization and may trigger inflammatory responses [3,8,9]. Immune response, in addition to osseointegration, significantly influences the success of implants, with macrophages playing a crucial role in regulating implant-associated bone immunity and the subsequent healing processes. Upon implantation, macrophages rapidly migrate to the material’s surface, where their polarization into distinct phenotypes is guided by the material’s physical and chemical properties. These macrophages typically adopt either a pro-inflammatory M1 phenotype or a pro-regenerative M2 phenotype [10]. While transient M1 activation is necessary for initial wound healing and pathogen clearance, excessive M1 activation leads to chronic inflammation, excessive fibrosis, and ultimately implant failure through fibrous encapsulation. On the other hand, M2 polarization is essential for successful angiogenesis, tissue repair, and stable osseointegration, as M2 macrophages secrete anti-inflammatory cytokines, growth factors, and matrix remodeling enzymes [11].

Recent studies have demonstrated that surface features modulate macrophage integrin expression profiles and cytokine secretion patterns, with specific topographical cues driving either pro-inflammatory or anti-inflammatory responses [11,12,13,14,15]. Beyond topography, various titanium-based surface chemical states have been engineered to modulate macrophage behavior. Graphene-oxide-modified TiO_2_ nanotubes reduce reactive oxygen species and dampen pro-inflammatory cytokine release while favoring M2 polarization [16]. Hydrogel coatings and H_2_S-releasing multilayer systems on titanium similarly promote a controlled M1 → M2 transition, improving bone–implant integration and vascularization [17,18]. Furthermore, research on polished titanium and nanostructured surfaces has revealed the remarkable immunomodulatory potential of anatase TiO_2_. For instance, honeycomb-like anatase with roughly 90 nm features has been shown to induce M2 macrophage polarization through RhoA/Rho–associated protein kinase signaling pathways [19]. Similarly, anodized TiO_2_ nanotubes exhibited size-dependent immunomodulatory effects, with about 30 nm tubes favoring M2c polarization while about 80 nm tubes inducing M1 activation [20]. Additional studies indicate that anatase nanotubes facilitate M2 polarization via ERK1/2 and PI3K/AKT signaling pathways [21], suppress NF-κB and MAPK activation [22], and reprogram macrophage metabolism through AMPK-mediated inhibition of glycolysis [23].

Despite these promising findings, there is currently an insufficient body of published research examining the effects of anatase coatings on local macrophage responses, particularly when applied to clinically relevant micro-rough SLA substrates. A thorough understanding of the influence of anatase-coated SLA surfaces on macrophage polarization is imperative, as existing knowledge of their effects is limited. The incorporation of a micro-nanostructured anatase coating may hold considerable potential to modulate early macrophage-mediated immune responses, which are integral to regulating osseointegration kinetics and maintaining the long-term stability of implants. In our recent work, we showed that depositing an anatase TiO_2_ layer on clinically relevant SLA titanium improves surface wettability/surface free energy and supports osteoblast viability and osteogenic function in vitro [24]. Building on this foundation, the present study examines whether the same micro–nano anatase-modified SLA surface also modulates macrophage responses and elicits measurable immunomodulatory effects. Therefore, the objective of this study was to systematically and comparatively investigate primary human monocyte-derived M0, M1, and M2 macrophages (MDMs) cultured on anatase-coated SLA titanium, conventional SLA, machined titanium and control coverslip surfaces and to determine whether the anatase coating maintains cytocompatibility while modulating the balance between pro-inflammatory M1 and regenerative M2 phenotypes. MDMs were chosen as a physiologically relevant in vitro model for studying early innate immune responses to biomaterials. MDMs more closely reflect tissue macrophage behavior than immortalized monocytic cell lines (e.g., THP-1), and their use allows inclusion of inter-donor variability, which is important for translational relevance. Using a comprehensive analysis combining a CCK-8 assay, confocal laser scanning microscopy (CLSM), scanning electron microscopy (SEM), ELISA, and qRT-PCR, we evaluate macrophage metabolic activity and morphology, surface marker expression, cytokine secretion and gene expression profiles. This multi-level analysis was designed to determine whether anatase-coated SLA surfaces modulate macrophage behavior beyond that observed for conventional SLA surfaces, thereby providing insight into their immunological safety and potential functional advantages. Finally, these findings are expected to provide crucial insights into the immunomodulatory potential of anatase-coated SLA surfaces and to support the development of next-generation implant surface modifications.

## 2. Materials and Methods

### 2.1. Sample Preparation

Round grade 4 titanium discs with three distinct surface modifications were investigated. The first group consisted of polished (Ti-machined) samples, with dimensions of 12 mm in diameter and 2 mm in thickness, characterized by a low surface roughness (Sa = 0.078 ± 0.006 µm) and a moderately hydrophilic surface wettability (water contact angle = 88.9 ± 5.9°). The second group was generated by sandblasting and acid-etching of the Ti-machined surfaces (Ti-SLA), resulting in a microrough topography (Sa = 1.55 ± 0.06 µm) and a hydrophobic surface behavior (water contact angle = 114.6 ± 0.8°). The third group received an anatase coating using a unipolar pulsed DC sputtering process (Ti-SLA-anatase), as previously described and characterized [24,25]. This surface exhibited a hierarchical topography consisting of multi-globular microrough microstructures (Sa = 1.88 ± 0.43 µm) decorated with polygonal nanoscale features and exhibiting markedly increased wettability, characterized by a near-superhydrophilic water contact angle (3.8 ± 5.4°). Surface roughness and static water contact angle values are reported as mean ± standard deviation and were obtained from prior physicochemical characterization of the same surfaces (n = 5 per surface) [24]. All discs were provided by Medentis Medical GmbH, Bad Neuenahr-Ahrweiler, Germany. Tissue-culture–treated coverslips were used as a control (no titanium surface) across all assays. Before all cell-based assays, the discs were cleaned ultrasonically in 70% ethanol for 5 min, immersed in fresh 70% ethanol for sterilization under sterile conditions for 15 min, and air-dried for 1 h under laminar airflow in a biosafety cabinet.

### 2.2. Isolation and Differentiation of Blood Monocyte-Derived Macrophages

Human peripheral blood was obtained from healthy donors under a protocol approved by the Ethics Committee of the University of Tübingen’s medical faculty (ethical approval: 286/2021 BO). Peripheral blood mononuclear cells (PBMCs) were isolated using density gradient centrifugation from three independent healthy donors [26]. Each independent experiment (biological replicate) was performed using cells from a single donor, and donor material was never pooled. MDMs were generated from PBMCs as described previously [27]. Briefly, monocytes were enriched by incubation in Monocyte Attachment Medium (PromoCell, Heidelberg, Germany; Cat No. C-28051) for 90 min at 37 °C. Non-adherent cells were removed by washing with PBS (Fisher Scientific, Schwerte, Germany; Cat No. 14190-169). Attached monocytes were cultured for differentiation into macrophages in RPMI 1640 medium (Fisher Scientific, Schwerte, Germany; Cat No. 15303541), supplemented with 10% heat-inactivated fetal bovine serum (FBS; Bio&Sell, Nürnberg, Germany; Cat No. FBS.S.0615), 1% penicillin/streptomycin (Gibco, Grand Island, NY, USA; Cat. No. 15140122), and 10 ng/mL macrophage colony-stimulating factor (M-CSF; PromoCell, Heidelberg, Germany; Cat. No. 300-25-10UG). Cells were differentiated for 6 days at 37 °C with 5% CO_2_, and the medium was refreshed every 2 days.

### 2.3. Cultivation and Polarization of MDMs on Different Surfaces

On day 6 of differentiation, MDMs were detached using Accutase (Merck, Darmstadt, Germany; Cat. No. SCR005) for 30 min at 37 °C and collected by centrifugation at 300× *g* for 10 min. Cells were resuspended in complete medium and seeded at a density of 1.6 × 10^5^ cells per 1 mL of medium onto the respective titanium discs and control coverslip placed in 24-well non-treated cell culture plates. Polarization was initiated post-seeding, wherein macrophages were cultured under specific polarization conditions for 48 h. M1 polarization was induced by supplementation with IFN-γ (50 ng/mL; Invitrogen, Carlsbad, CA, USA; Cat. No. RIFNG100) and LPS (10 ng/mL; Sigma Aldrich, St. Louis, MO, USA; Cat. No. L4391), whereas M2 polarization was induced using IL-4 and IL-13 (20 ng/mL each; Miltenyi Biotec, Bergisch Gladbach, Germany; Cat. No. 200-04, Cat. No. 200-13), as previously described [27]. M0 macrophages were maintained in complete medium without polarization agents. All cultures were incubated for 48 h at 37 °C in a humidified atmosphere containing 5% CO_2_. Macrophage polarization status was assessed using complementary readouts, including immunofluorescence staining (CLSM), cytokine quantification (ELISA), and gene expression analysis (qRT-PCR), using subtype-associated markers for M1 and M2 phenotypes. The selected markers were chosen based on their reported polarization specificity in human monocyte-derived macrophages [27,28,29,30] and were further supported by prior internal optimization experiments in our laboratory.

### 2.4. Assessment of Metabolic Activity

The relative metabolic activity of M0-, M1-, and M2-MDMs was determined using a Cell Counting Kit-8 (CCK-8; Dojindo Laboratories, Kumamoto 861-2202, Japan; Cat. No. CK04-13) after 48 h of incubation on different surfaces and in three biological replicates per surface condition. Following incubation, cells were washed with PBS and incubated in 500 µL of complete culture medium containing 10% (*v*/*v*) of CCK-8 reagent for 1 h at 37 °C. The absorbance was measured at 450 nm using a microplate reader (Infinite F50; Tecan Austria, Grödig, Austria) and served as a quantitative indicator of cellular metabolic activity.

### 2.5. Cytokine Secretion Analysis

Culture supernatants were collected after 48 h of macrophage incubation on the test surfaces and centrifuged at 300× *g* for 5 min to remove cellular debris. The concentrations of tumor necrosis factor-alpha (TNF-α, as an M1-associated cytokine) and C-C motif chemokine ligand 13 (CCL13, as an M2-associated chemokine) were quantified using ELISA kits (Thermo Fisher Scientific, Carlsbad, CA, USA; TNF-α ELISA kit; Cat. No. KHC3011, CCL13 ELISA kit; Cat. No. EHCCL13), following the manufacturer’s protocols. Absorbance was recorded at 450 nm using a microplate reader, and cytokine levels were calculated by interpolation from standard curves. To account for variability in cell number, cytokine concentrations were normalized to total protein content in each well, measured using the Pierce BCA Protein Assay Kit (Thermo Fisher Scientific, Carlsbad, CA, USA; Cat. No. 23227). Cytokine secretion was analyzed using three independent replicates.

### 2.6. Cell Morphology Analysis Using CLSM and SEM

After 48 h of incubation on test surfaces, M0-, M1-, and M2-MDMs were fixed with 3% paraformaldehyde (Sigma Aldrich Co., Steinheim, Germany; Cat. No. P6148) for 10 min at room temperature (RT), permeabilized with 0.2% Triton X-100 (Merck, Darmstadt, Germany; Cat. No. X100) in PBS for 10 min, and washed with PBS. Samples were blocked in 1% bovine serum albumin (BSA; VWR International, Leuven, Belgium; Cat No. 422361V) and 0.05% Tween-20 (Roth, Karlsruhe, Germany; Cat. No. 9127.1) in PBS for 30 min. Cytoskeletal staining was performed using 5 µg/mL phalloidin–FITC (Sigma Aldrich, Saint Louis, MI, USA; Cat. No. P5282) in blocking buffer for 1 h, followed by nuclear staining with 10 µM DRAQ5 (Miltenyi Biotec, Bergisch Gladbach, Germany; Cat No. 130-117-344) for 15 min at RT. Imaging was conducted using a confocal laser scanning microscope (CLSM; Leica TCS SP5, Leica Microsystems, Mannheim, Germany). 

For SEM analysis, MDMs were fixed overnight in 2% (*v*/*v*) glutaraldehyde (Carl Roth, Karlsruhe, Germany; Cat. No. 4157.1), dehydrated via ascending ethanol concentrations, and critical point dried (E3100, Quorum, Darmstadt, Germany). Subsequently, samples were sputter-coated (SCD 050, Baltec, Lübeck, Germany) with a thin layer of Au-Pd and imaged at 5 kV accelerating voltage (SEM; LEO 1430, Zeiss, Oberkochen, Germany). All imaging parameters were kept constant across groups, and Images were captured from three random areas per sample.

### 2.7. Cell Surface Marker Analysis Using CLSM

Immunofluorescence staining for macrophage polarization markers was performed after 48 h of culture in all groups. Cells were fixed as outlined in Section 2.6, and non-specific binding sites were blocked in PBS containing 1% BSA and 0.1% Tween 20 for 30 min. Cells were then incubated with either mouse anti-human CCR7 antibody (M1 marker; 10 µg/mL, Clone 150503, R&D Systems, Minneapolis, MN, USA; Cat. No. MAB197-100) or rabbit anti-human CD209 antibody (M2 marker; 1:400, Clone D7F5C, Cell Signaling Technology, Danvers, MA, USA; Cat. No. 13193S) for 60 min at RT. After washing with PBS containing 0.1% Tween 20, cells were incubated with the corresponding secondary antibodies (Alexa Fluor 488 goat anti-mouse IgG, 5 µg/mL; Rockford, IL, USA, Cat No. A32723 or Alexa Fluor 488 goat anti-rabbit IgG, 1:200; Abcam, Waltham, MA, USA; Cat. No. ab150077) for 60 min in the dark at RT. Nuclei were counterstained with DRAQ5. Image acquisition was performed using the CLSM setup described in Section 2.6. Fluorescence intensities of CCR7 and CD209 cell surface markers were quantified from three randomly selected fields of view per disc using ImageJ (v1.53a), obtained from two independent biological replicates.

### 2.8. Quantitative Real-Time PCR Analysis

Following incubation on test surfaces for 3 h (for TNF-α and IL-1β) or 48 h (for all other genes), total RNA was extracted from M0-, M1-, and M2-MDMs using the RNeasy Micro Kit (Qiagen, Hilden, Germany; Cat. No. 74004) according to the manufacturer’s protocol. cDNA synthesis was performed using 350 ng of RNA with the LunaScript RT SuperMix (New England Biolabs, Ipswich, MA, USA; Cat. No. M3010X). qRT-PCR was carried out using the Luna Universal qPCR Master Mix (New England Biolabs, Ipswich, MA, USA; Cat. No. M3003E) on a QuantStudio 3 Real-Time PCR System (Applied Biosystems, Thermo Fisher Scientific, Waltham, MA, USA). The cycling protocol included an initial denaturation at 95 °C for 3 min, followed by 40 cycles at 95 °C for 15 s and 55 °C for 30 s with a subsequent melting curve analysis performed at the end of each run to verify amplification specificity (melting curves shown in Figure S1). Primer sequences for target genes and the housekeeping gene GAPDH are listed in Table 1. All primers were synthesized by standard solid-phase DNA synthesis and purified by HPLC (Ella Biotech GmbH, Fürstenfeldbruck, München, Germany). Relative gene expression was calculated using the comparative Ct 2^(−ΔΔCT) method as described by Livak and Schmittgen [31], normalized to GAPDH, with M0-MDMs cultured on coverslip serving as the reference condition. Gene expression analyses were conducted with three independent biological replicates. For CD209, one replicate was excluded due to an unsuccessful assay run; consequently, CD209 results are based on two independent biological replicates.

### 2.9. Statistical Analysis

All statistical analyses were performed using GraphPad Prism version 10.1.1 (GraphPad Software, San Diego, CA, USA). Data normality was assessed using the Shapiro–Wilk test. For normally distributed datasets, one-way ANOVA followed by Sidak’s multiple comparisons test was applied. For non-normally distributed datasets, the Friedman test followed by Dunn’s multiple comparisons test was used. A *p*-value < 0.05 was considered statistically significant. Statistical significance is indicated as follows: * *p* < 0.05, ** *p* < 0.01, *** *p* < 0.001, and **** *p* < 0.0001. Data are expressed as mean ± standard error of the mean (SEM).

## 3. Results and Discussion

### 3.1. Macrophage Viability and Morphology on Different Titanium Surfaces

**Figure 1:**
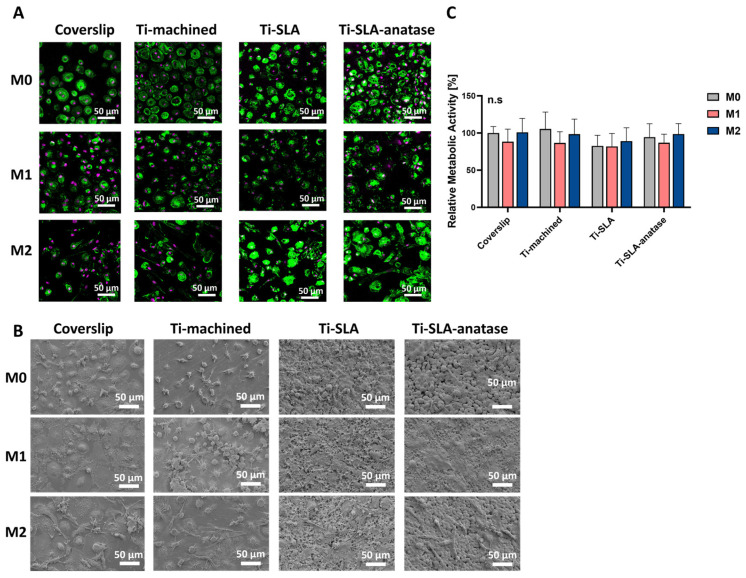
Behavior of M0-, M1-, and M2-MDMs on differently modified titanium Surfaces. (**A**) Representative CLSM images showing M0-, M1-, and M2-MDMs on coverslip, Ti-machined, Ti-SLA, and Ti-SLA-anatase surfaces after 48 h of incubation. The cytoskeleton (F-actin) was stained with FITC-Phalloidin (green), and nuclei were stained with DRAQ5 (purple). (**B**) Representative SEM images of M0-, M1, and M2 macrophages on coverslip and titanium surface modifications. (**C**) Metabolic activity was assessed using the CCK-8 assay, with values normalized to coverslip M0 (set as 100%). Data are presented as mean ± SEM. n = 3. No significant differences were observed between groups (Friedman test with Dunn’s multiple comparisons test).

The behavior of M0-, M1-, and M2-polarized MDMs on the different titanium surfaces as well as on the coverslip control was evaluated by assessing cell morphology and metabolic activity. CLSM and SEM imaging after 48 h provided insight into macrophage morphology (Figure 1A,B). M0-MDMs incubated on all four surfaces exhibited a predominantly round to slightly polygonal shape with moderate spreading and a clear cortical F-actin organization, consistent with the morphology typically described for non-polarized macrophages [13,27]. On all surfaces, M1-MDMs displayed a larger, flatter “pancake-like” morphology with relatively rigid cell edges and increased membrane ruffling and filopodia, in line with their pro-inflammatory, classically activated phenotype. In contrast, M2-MDMs, which are associated with anti-inflammatory and tissue-healing functions, showed a more elongated or spindle-shaped morphology with pronounced cell body polarization, which was visible on all three titanium surface modifications as well as on the control surface [32]. Metabolic activity, measured using the CCK-8 assay, showed no significant differences among MDM subtypes cultured on coverslip controls, Ti-machined, Ti-SLA, and Ti-SLA-anatase surfaces after 48 h (Figure 1C). Furthermore, the viability of polarized M0, M1, and M2 macrophages was comparable between surfaces. This indicates that all three titanium surface types supported comparable macrophage viability and that the polarization state did not markedly affect overall metabolic activity within the investigated period.

Overall, these observations indicate that macrophage morphology in this model was primarily dictated by polarization state, with titanium surface modification exerting only subtle additional effects. On Ti-SLA-anatase surfaces, macrophages showed slightly greater spreading and more pronounced cytoskeletal organization compared with Ti-SLA; however, this observation is qualitative and was not subjected to morphometric quantification. This may relate to the combined micro-/nano-roughness and increased wettability of the modified surface, which are known to enhance integrin-mediated adhesion and cytoskeletal remodeling [13,15]. However, the superimposed nanoscale anatase features did not substantially override the influence of the underlying SLA micro-roughness within the 48 h timeframe. Taken together, the preserved metabolic activity and the absence of obvious cell detachment or gross morphological signs of damage in CLSM and SEM analyses suggest that none of the tested surfaces induced acute cytotoxic effects during the 48 h culture period. Accordingly, cytocompatibility conclusions in this study are based on the combined CCK-8 and imaging readouts, and no additional dedicated viability assay (e.g., Annexin V/PI or trypan blue exclusion) was performed.

### 3.2. Macrophage Polarization Profiles on Different Titanium Surfaces

**Figure 2:**
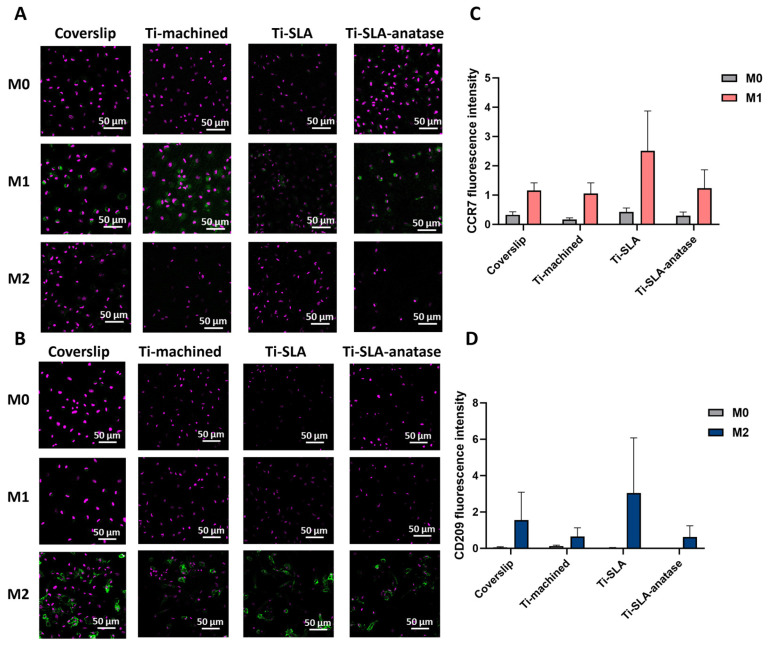
CLSM analysis of macrophage surface markers CCR7 (M1) and CD209 (M2) on differently modified titanium surfaces. Representative confocal images of macrophages cultured for 48 h on coverslip, Ti-machined, Ti-SLA, and Ti-SLA-anatase surfaces under M0, M1, and M2 polarization conditions. Cells were stained for the M1 marker CCR7 (**A**); (green) and the M2 marker CD209 (**B**); (green), with nuclear counterstaining (purple). CCR7 and CD209 staining was performed in parallel in separate wells (no co-staining). Quantification of CCR7 (**C**) and CD209 (**D**) immunofluorescence intensity. Mean fluorescence intensity was quantified in ImageJ from three randomly selected fields of view per disc. Data are presented as mean ± SEM from two independent biological replicates (n = 2); results are shown descriptively.

CLSM analysis of CCR7 (M1 marker; Figure 2A) and CD209 (M2 marker; Figure 2B) expression revealed clear polarization-dependent expression patterns, with all surfaces supporting robust marker induction after polarization. M1 macrophages displayed high CCR7 signal, whereas M2 macrophages expressed strong CD209 signal on all surfaces. Under M0 conditions, only faint baseline expression of both markers was detectable, consistent with their non-polarized status [27,33,34]. Quantitatively, CCR7 and CD209 fluorescence intensities showed subtle surface-dependent differences, with Ti-SLA exhibiting slightly higher marker intensities than coverslip, Ti-machined, and Ti-SLA-anatase. However, these differences appeared modest and were not analyzed statistically due to the limited number of biological replicates (n = 2). Instead, intensity trends are presented descriptively to illustrate polarization-dependent patterns across surfaces. While SLA micro-roughness may mildly amplify receptor expression, the anatase coating did not visually appear to induce additional shifts beyond those observed with the SLA substrate.

Previous studies have demonstrated that surface topography and chemistry can influence macrophage polarization states, inducing changes in cell morphology, adhesion, and mechanotransductive signaling [12,13,14,15,19]. However, our data show only modest, qualitatively assessed differences in CCR7 and CD209 expression between surfaces, suggesting that in this experimental setting the imposed cytokine polarization (M0, M1, M2) is the dominant driver of phenotype. On top of an already micro-rough SLA substrate, the sputtered anatase nanocoating does not measurably further modulate CCR7 or CD209 expression within 48 h.

One possible explanation is that nanoscale features might meet specific dimensional and morphological criteria to effectively influence macrophage polarization, rather than the mere presence of a nanostructure [19,23,35]. For example, Wang et al. demonstrated that different titanium nanotube diameters produced distinct polarization patterns, with certain nanotopographies favoring M1 while others promoted M2 phenotypes and concurrently enhancing downstream osteogenesis and osseointegration [23,35]. Similarly, Zhu et al. showed that TiO_2_ honeycomb nanotopographies, particularly those with roughly 90 nm features, promoted M2-associated marker expression (e.g., CD206), increased IL-4/IL-10 and BMP-2 secretion, and enhanced osteogenesis both in vitro and in vivo [19]. Additionally, the limited response observed could reflect the short duration of the study, which may not allow enough time for surface-mediated macrophage reprogramming to fully occur.

**Figure 3:**
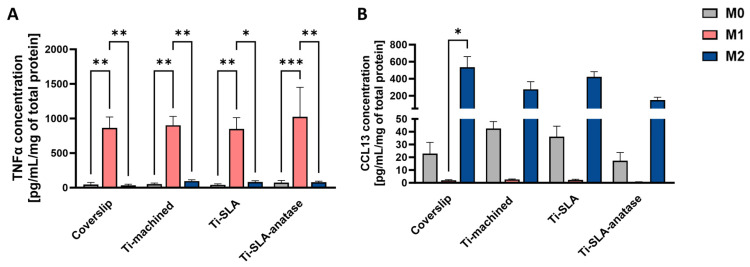
Cytokine secretion of M0-, M1-, and M2-MDMs cultured on coverslip control and different titanium implant surfaces for 48 h. Secretion of TNF-α (**A**) and CCL13 (**B**) in cell culture supernatants was quantified by ELISA and expressed as pg/mL/mg total protein. Bars represent mean ± SEM (n = 3). Statistical significance was determined using one-way ANOVA followed by Sidak’s multiple comparisons test for normally distributed data (TNF-α). For non-normally distributed datasets, the Friedman test followed by Dunn’s multiple comparisons test was applied (CCL13). * *p* < 0.05, ** *p* < 0.01, *** *p* < 0.001.

Cytokine secretion data indicated that macrophage polarization state, rather than titanium surface chemistry or topography, was the primary driver of both pro-inflammatory and M2-associated secretory output after 48 h (Figure 3). M1 macrophages secreted high levels of TNF-α across all surfaces, significantly higher than those produced by M0 and M2 subtypes on the corresponding surface. However, the level of TNF-α secretion was comparable on Ti-machined, Ti-SLA, and Ti-SLA-anatase. This indicates that neither the micro-rough SLA treatment nor the anatase coating noticeably affected the magnitude of classical M1 activation (Figure 3A).

M2 macrophages secreted higher amounts of CCL13 than M0 and M1 cells, indicating that none of the tested surfaces interfered with cytokine-driven induction of an anti-inflammatory phenotype (Figure 3B). However, the levels of CCL13 were comparable across all titanium surfaces, suggesting that surface properties do not influence macrophage responses within this experimental setting. Overall, these findings reinforce that macrophage polarization state is the dominant factor shaping TNF-α and CCL13 secretion, while the titanium surface properties examined in this study do not demonstrate modulatory effects under the tested conditions. The absence of strong surface-dependent modulation of cytokine secretion contrasts with studies of highly ordered anatase nanotubes, which reported suppression of pro-inflammatory cytokines via NF-κB and MAPK pathway inhibition [22]. This discrepancy may reflect that the specific size and morphology of the anatase nanocrystalline structures in our system do not fall within the range required to elicit a pronounced immunomodulatory response, or may indicate that cytokine modulation requires longer culture periods or specific nanoscale topographical dimensions not present in our specific coating.

**Figure 4:**
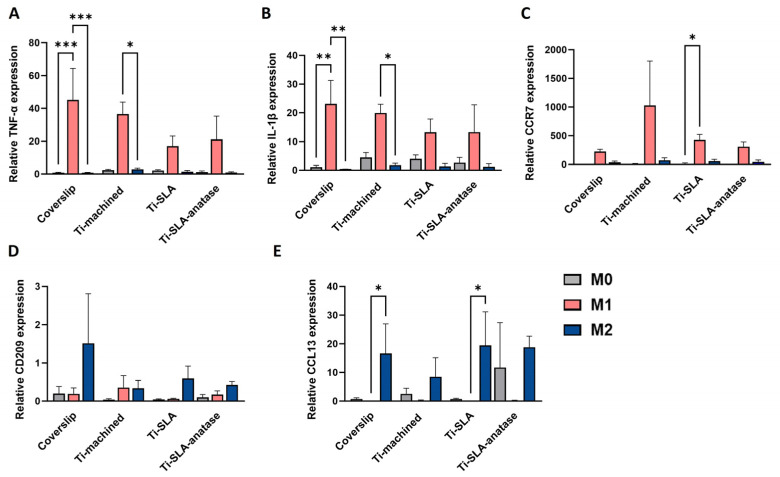
Gene expression profiles of M0-, M1-, and M2-MDMs cultured on different titanium implant surfaces and control coverslip. Relative gene expression of TNF-α (**A**), IL-1β (**B**), CCR7 (**C**), CD209 (**D**), and CCL13 (**E**) in macrophages cultured on coverslip, Ti-machined, Ti-SLA, and Ti-SLA-anatase surfaces for 3 h (TNF-α, IL-1β) or 48 h (all other markers), analyzed by qRT-PCR. Bars represent mean ± SEM (n = 2 for CD209 and n = 3 for all other genes). Statistical significance was determined using one-way ANOVA followed by Sidak’s multiple comparisons test for normally distributed data. For non-normally distributed datasets, the Friedman test followed by Dunn’s multiple comparisons test was applied. * *p* < 0.05, ** *p* < 0.01, *** *p* < 0.001.

To further investigate how titanium surfaces influence macrophage activation at the transcriptional level, we analyzed expression of the M1-associated markers TNF-α, IL-1β, and CCR7, and the M2-associated markers CD209 and CCL13 by qRT-PCR (TNF-α and IL-1β at 3 h, all other genes at 48 h; Figure 4). Across all three surfaces, M0-MDMs showed low transcript levels for these markers, consistent with their non-polarized state. M1-MDMs exhibited a marked upregulation of TNF-α, IL-1β, and CCR7 relative to M0 and M2 macrophages on each surface, confirming classical activation in agreement with their phenotype (Figure 4A–C). Although TNF-α, IL-1β, and CCR7 expression in M1 cells appeared numerically lower on Ti-SLA and Ti-SLA-anatase compared to Ti-machined, these differences were not statistically significant, indicating that neither SLA micro-roughness nor the anatase coating induces a pro-inflammatory polarization switch in macrophages. As expected, M2-MDMs showed the highest expression of the M2-associated markers CD209 and CCL13 compared with M0 and M1 macrophages (Figure 4D,E). For CD209 and CCL13, M2 cells on Ti-SLA-anatase and Ti-SLA tended to exhibit higher transcript levels compared to Ti-machined, whereas no significant surface-dependent differences were detected. These results again reinforce that polarization state, rather than surface chemistry or topography, was the dominant determinant of M1/M2 marker transcription under the present study conditions.

Taken together, the three titanium surface modifications (Ti-machined, Ti-SLA, Ti-SLA-anatase) exerted only modest, mostly non-significant modulatory effects. Importantly, none of the surfaces induced evident cytotoxicity or exaggerated M1 activation, indicating that both the conventional SLA and the anatase-coated SLA surfaces are immunologically well tolerated under the conditions tested. In addition, inclusion of a coverslip control provided a reference for standard culture, surface-free conditions. Across readouts, macrophage metabolic activity and the expected polarization-dependent marker/cytokine patterns on titanium were broadly comparable to the coverslip condition within 48 h, supporting the interpretation that the imposed M0/M1/M2 polarization cues were the dominant drivers of phenotype in this model. The magnitude of effects in the current study appears lower than the immunomodulation reported for highly structured nanotopographies such as TiO_2_ nanotube arrays and honeycomb-like anatase structures [19,23,35], suggesting that strong macrophage reprogramming likely requires nanoscale features with specific dimensional and morphological parameters. The hierarchical nature of the Ti-SLA-anatase surface in our study may further dampen additional effect of the nanotexture. SLA micro-roughness is known to exert strong control over protein adsorption, cell attachment, and mechano-transduction, and hydrophilic micro-rough titanium has been reported to bias macrophages toward a less inflammatory, more pro-osteogenic phenotype compared with smooth or hydrophobic surfaces [11,13]. In such a context, it is plausible that the robust micro-topography dominates cell–material interactions, while the superimposed nanostructure contributes only incrementally and therefore most likely remains below the detection threshold in a short-term in vitro assay.

Previous studies indicate that pro-inflammatory M1 responses emerge rapidly, while markers associated with M2 responses take several days to develop fully in both in vitro and in vivo settings [14,17,18]. Accordingly, the consistently low and non-elevated inflammatory readouts on Ti-SLA-anatase in our 48 h model support the view that this surface does not exacerbate macrophage-mediated inflammation, even if longer-term M2 enhancement could not be captured here. From the perspective of functional biomaterial design, the current findings still offer valuable insights. First, our findings indicate that applying a nanoscale anatase coating on a well-established clinically successful microrough SLA surface does not introduce an undesirable pro-inflammatory macrophage response, which is an important consideration. Second, the mild tendencies toward M2-associated markers on the rough surfaces, though not statistically robust, are consistent with the broader literature linking micro-/nano-rough hydrophilic titanium to more pro-healing immune microenvironments [11,12,13,14,15]. When considered together with our previous osteogenic data on the same surface [24], the present findings raise the hypothesis that anatase-coated SLA titanium may support osseointegration by combining improved osteoblast responses with an immunologically neutral macrophage profile; however, future studies will be required to test this hypothesis directly.

## 4. Conclusions

In this study, we explored the immunomodulatory potential of three different titanium surfaces using primary human monocyte-derived macrophages. Overall, the surfaces showed high cytocompatibility, and macrophages of all subtypes adhered and survived well on all surface modifications without signs of cytotoxicity. Across all experiments, the macrophage polarization state itself turned out to be the dominant factor shaping their behavior, while the titanium surface modifications only caused rather subtle differences. The anatase-coated SLA surface did not lead to significant changes in M1 or M2 polarization within the 48 h period compared to Ti-machined and Ti-SLA. We note that the 48 h observation window reflects only the early phase of macrophage activation and does not capture long-term immune responses or later stages of osseointegration. Within the limitations of this study, these findings indicate that anatase-coated SLA surfaces are immunologically well tolerated and provide a safe basis for further optimization. Future studies with longer culture periods, a higher number of biological replicates, macrophage–osteoblast co-cultures, and in vivo validation are required to determine whether more subtle pro-regenerative immune effects emerge under physiological conditions.

## Figures and Tables

**Table 1:** Primer sequences used in qRT-PCR.

Primer Name	Forward Primer Sequence	Reverse Primer Sequence
GAPDH	5′-GAGTCAACGGATTTGGTCGT-3′	5′-TTGATTTTGGAGGGATCTCG-3′
CCR7	5′-TGGTGATCGGCTTTCTGGTC-3′	5′-CACCTTGATGGCCTTGTTGC-3′
CD209	5′-GGAGCAGAACTTCCTACAGC-3′	5′-CAACGTTGTTGGGCTCTCCT-3′
CCL13	5′-ATCTCCTTGCAGAGGCTGAA-3′	5′-ACTTCTCCTTTGGGTCAGCA-3′
TNFα	5′-GCTGCACTTTGGAGTGATCG-3′	5′-TCACTCGGGGTTCGAGAAGA-3′
IL-1β	5′-AGCTGATGGCCCTAAACAGA-3′	5′-TGGTGGTCGGAGATTCGTAG-3′

## Data Availability

Data are contained within the article.

## Supplementary Materials

The following supporting information can be downloaded at: https://www.mdpi.com/article/10.3390/jfb17030111/s1. Supplementary Figure S1: Melting-curve analysis of qRT-PCR amplicons. Melting curves for each primer set showed a single dominant peak, indicating specific amplification. Melt curves are shown as the derivative of normalized reporter fluorescence (−dRn/dT) versus temperature. (A) GAPDH, (B) TNF-α, (C) IL-1β, (D) CCL13, (E) CCR7, (F) CD209.
