# Supplementary material for: Influence of Micro-Nanostructured Anatase-Coated SLA Titanium on Macrophage Behavior
Source: J Funct Biomater. 2026 Feb 25;17(3):111. doi: 10.3390/jfb17030111 (PMC13026796; doi:10.3390/jfb17030111)

**Supplementary Figure S1. Melting-curve analysis of qRT-PCR amplicons.** Melting curves for each primer set showed a single dominant peak, indicating specific amplification. Melt curves are shown as the derivative of normalized reporter fluorescence (−dRn/dT) versus temperature. (A) GAPDH, (B) TNF-α, (C) IL-1β, (D) CCL13, (E) CCR7, (F) CD209.

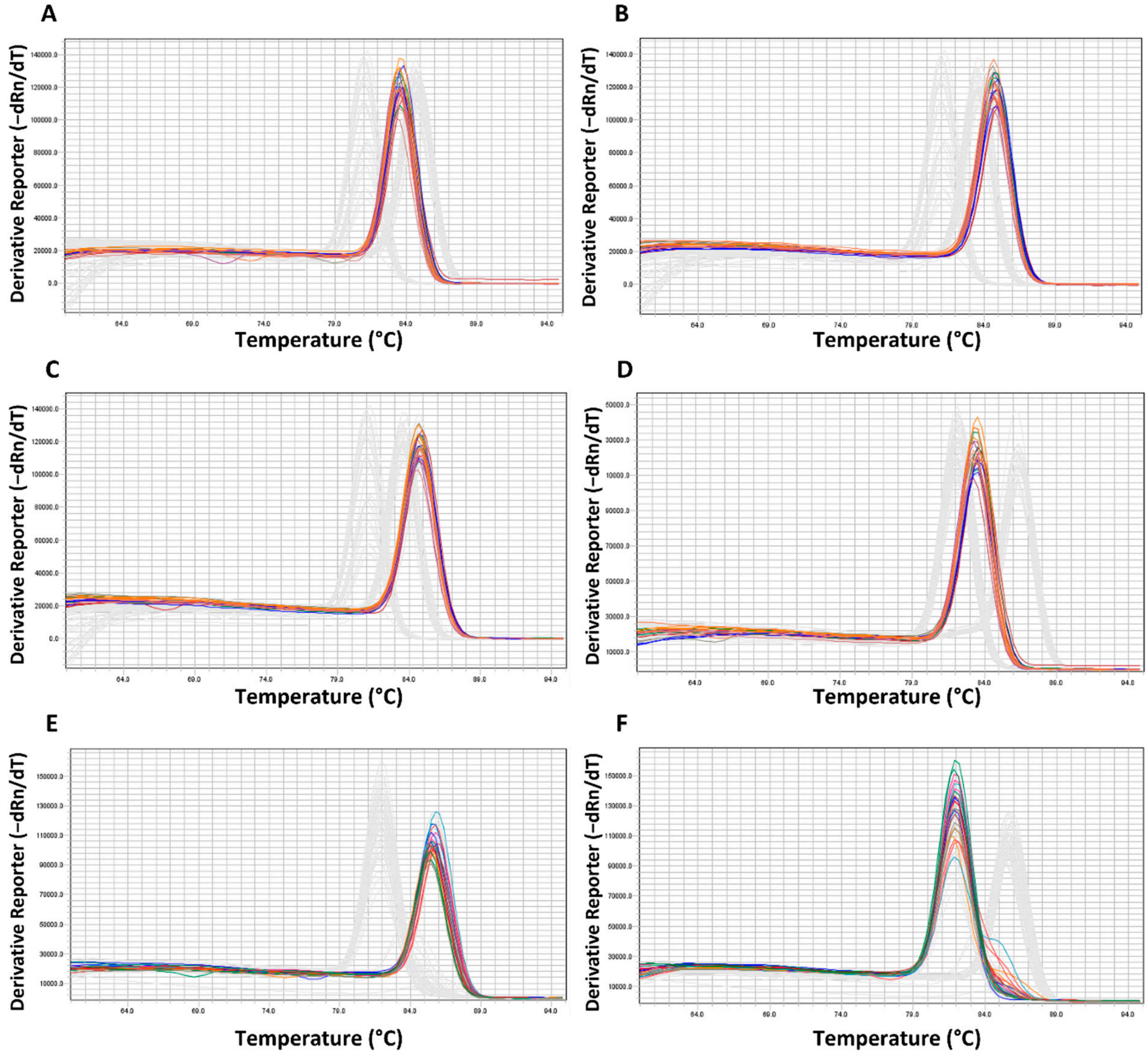

Supplement: Supplementary file 1 [file jfb-17-00111-s001.zip › jfb-4070280-supplementary.pdf]
